# Screening for distress, related problems and perceived need for psycho-oncological support in head and neck squamous cell carcinoma (HNSCC) patients: a retrospective cohort study

**DOI:** 10.1186/s12885-021-08236-2

**Published:** 2021-04-30

**Authors:** V. Kunz, G. Wichmann, A. Lehmann-Laue, A. Mehnert-Theuerkauf, A. Dietz, S. Wiegand

**Affiliations:** 1 Department of Otolaryngology, Head and Neck Surgery, University Medical Center Leipzig, Leipzig, Germany; 2 Department of Medical Psychology and Medical Sociology, University Medical Center Leipzig, Leipzig, Germany

**Keywords:** Head and neck squamous cell carcinoma, Distress thermometer, Psycho-oncological care

## Abstract

**Background:**

In different cancer entities, several studies have shown the adverse effects of cancer on mental health, psychological well-being and the increased risk of high emotional distress in cancer patients. This study aims to analyze psychosocial distress levels and their relationship between sociodemographic parameters and selected items on the Distress Thermometer (DT) Problem List in head and neck squamous cell carcinoma (HNSCC) patients.

**Patients and methods:**

We assessed a total of 120 HNSCC patients using the Distress Thermometer (DT) Problem List. Distress scores (DTS) of 90 patients were available. A DTS of ≥ 5 on the visual analogue scale represents clinically relevant distress. Data analysis consisted of descriptive statistics, comparison of mean values for different DTS subcategories and correlation between DTS scores and parameters of tumor classification, sociodemographic variables and selected problems.

**Results:**

Distress was present in 57.7% of the sample, with a total of 52 patients with a DTS  ≥ 5. The mean DTS was 4.7 (SD 2.4). Patients with newly diagnosed HNSCC had significantly higher DTS. Distress levels were significantly associated with sadness, general worries, anxiety, nervousness, sleeping disorders, mouth sores and fever. Out of the total sample, 6 patients and out of these 6 individuals, 5 patients with a DTS ≥ 5 requested referrals to psycho-oncological service.

**Conclusion:**

High distress levels were common in HNSCC patients but only few patients desired psycho-oncological care. Addressing patients’ supportive care needs in routine clinical practice is essential to meet unmet needs of HNSCC patients and thus improve cancer care.

## Introduction

Patients with head and neck squamous cell carcinoma (HNSCC) represent a particular challenge from a psycho-oncological point of view. Compared to patients with other tumor entities, HNSCC patients have been found to show elevated levels of psychological distress including anxiety and depression, higher subjective burden of mental illness [1], mental disorders [2] as well as an increased suicide rate [3]. Therefore, adequate and timely assessment of psychosocial burden is relevant in HNC patients. In this context, the accreditation of HNC centers in Germany in compliance with requirements issued by the German Cancer Society requires the implementation of a qualified psycho-oncology program to provide psycho-oncological care for these patients. Past studies have pointed out the need for implementation of professional psycho-oncological treatment concepts into the standard therapy of these patients [4]. However, in most treating centers, psycho-oncological care is still provided on a voluntary basis only, based on the patients’ request. To obtain the latter, however, could be difficult in HNSCC patients, as many of them belong to an underprivileged group characterized by a rather low socio-economic status and a high prevalence of lifestyle factors linked to development of HNSCC and relapse including alcohol consumption and tobacco smoking. On the other hand, the group of patients with HPV-16 associated oropharyngeal squamous cell carcinoma (OPSCC), who are characterized by a very different socio-demographic profile than patients with HNSCC relating to smoking or alcohol, is presently sharply on the increase. HPV-related OPSCC is considered to be a separate disease with a causal relationship to HPV infection, different clinical characteristics and a better prognosis. HPV-positive disease also has been demonstrated to have a significant psychosocial impact on the patients [5].

In all, only about 30% of the patients identified as having significant psychological distress received a recommendation or referral over the course of their medical treatments [6, 7].

Using other diagnostic tools, a high willingness to participate in psycho-oncological intervention could already be shown for cancer patients over 60 years of age [8]. One instrument to assess psychological and psychosocial effects is the National Comprehensive Cancer Network (NCCN) Distress Thermometer (DT), which is widely used in cancer centers for screening [9]. According to receiver operator characteristics, the DT at a cut-off value of five is able to detect patients with a Hospital Anxiety Depression Scale score of ≥ 15 (that is often described being the gold standard for depression diagnosis) with a high sensitivity of 0.85 and moderate specificity of 0.69 [10]. Therefore, Tuinman et al. proposed the DT cut-off value of five for categorizing patients accordingly [10]. However, studies in lung cancer patients report a prevalence of distress according to a DT above five in 34.5% of non-small cell lung cancer (NSCLC) patients [11].

Distress is defined by the NCCN as “a multifactorial, unpleasant, emotional experience of a psychological, social, and/or spiritual nature that may interfere with the ability to cope effectively with cancer, its physical symptoms, and its treatment” (NCCN 2007). In HNSCC, patients’ pain and high levels of distress are reported rather early, before their further assessment and therapy is determined [12]. Furthermore, self-reported history of depression, family concerns, emotional concerns and physical concerns seem to be associated with high levels of distress [13]. For cancer patients, including patients with HNSCC, a psychosocial initial screening enables reported problems to be differentiated and targeted in a specific manner and thus offers a consultation service that is adjusted to these [11]. However, data on acceptance or more precisely, demand for psycho-oncological care, in patients with HNSCC are scarce.

The aim of the present study was to estimate the frequency of patients with high level of distress (DT score ≥ 5 [14–16]) among head and neck cancer patients and to analyze and compare the mean distress-levels associated with demographic characteristics and the demand for psycho-oncological care in a patient cohort with HNSCC.

## Patients and methods

### Statistical considerations and sample size

The calculation of our aimed sample size was performed on the basis of an expected rate of approximately 34.5% based on reported 34.5% in NCLC [11], 34.0% in breast cancer [14] and 35.0% in gynecologic cancer patients [15] with DT score ≥ 5 [14–16]. Presuming a DTS ≥ 5 frequency of 34.5% and a maximum error of ε = 10% in estimating the presence of distress among HNSCC patients demanding psycho-oncologic referral, a total of 87 questionnaires with full coverage of data would be required to answer the question about same frequency of distress. Given the high dropout observed in questionnaire-based assessments in one earlier HNSCC study reaching a maximum of 28% missing data within a particular item, we planned to accrue a sample of *n* = 120 patients.

### Study cohort

**Fig. 1:**
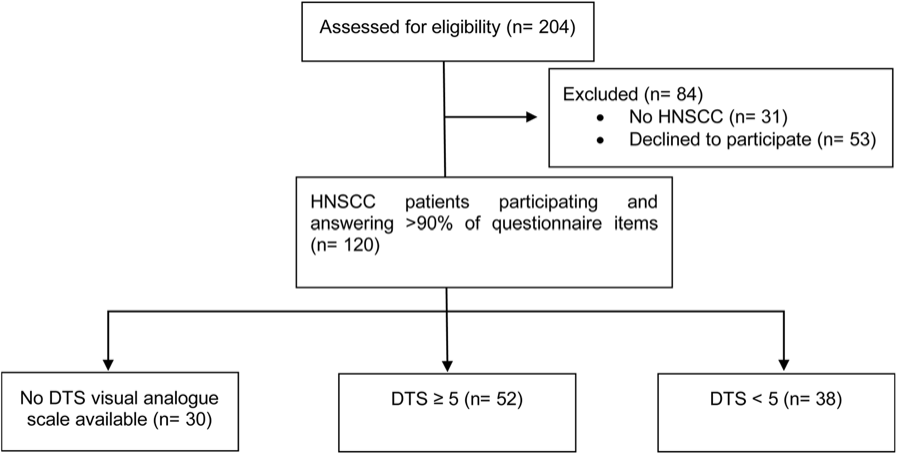
CONSORT Flow diagram for patient selection and study cohort

The study population was composed of 120 consecutively accrued HNSCC patients presented to the Department of Otolaryngology, Head and Neck Surgery for the assessment and management of their cancer from January 2018 to September 2020. The study was approved by our institutional review board (ethics committee of the Medical Faculty, vote 1786–15-01062015). After providing informed consent, the patients were asked to complete the DT and the problem list questionnaire on the day of admission as screening procedure for assessing the need for psycho-oncological intervention as recommended in the German S3-guideline for psycho-oncology. All participating patients had sufficient knowledge of the German language in order to complete the questionnaire on their own. According to the study protocol, we finished the enrollment after recording of the 120th patient in September 2020, and started retrospective analyses of questionnaires. Patient selection is shown in Fig. 1. The following patient data, which could influence psycho-oncological distress, were evaluated: sex, age, living with a partner, tumor site and tumor stage according to the TNM 8th edition.

### The distress thermometer

The Distress Thermometer (DT) is a brief, validated screening measure to identify the level of distress and the sources of distress [9]. We used the validated German adaptation [17]. The DT consists of a 11-point visual analogue scale ranging from 0 (no distress) to 10 (severe distress) to quantify the global level of distress experienced in the past week including the current day, as well as a standardized problem checklist containing 36 potential causes of distress (yes/no questions) that are grouped into 5 categories including physical problems, practical, family, emotional problems, and spiritual/religious concerns. The DT is recommended by the NCCN guidelines to be completed at pivotal time points during patients’ cancer treatment. Patients with a DTS of ≥ 5 on the visual analogue scale are considered to have significant distress necessitating psychosocial referral [18, 19]. All patients stated their demand for psycho-oncological referral in written form (“Do you wish that we organize an appointment with a psycho-oncologist for you?”, Yes/No). If” Yes” was checked, the physician responsible for the patient contacted the department for medical psychology in order to promptly enable first contact between a psycho-oncologist and the patient.

### Demographic parameters

The demographic parameters “Sex”, “Age”, “Living with a partner”, “Tumor site” and “Tumor stage” were chosen to investigate their potential influence on distress of HNSCC patients, as previous studies in other malignancies were able to show that distress was significantly higher in patients being female, being at a specific age group and having different tumor sites [20]. On the other hand, there is evidence in patients with, for instance bladder cancer, that emphasize the importance of the role of a supportive partner [21]. Regarding tumor stage, there is evidence in patients with gastric or ovarian cancer, that an advanced tumor stage is significantly associated with higher distress and also higher levels of anxiety and depression [22, 23]. Based on these findings in other malignancies, the relationship of these potential influencers of distress was analyzed in our cohort of HNSCC patients.

### Statistical analysis

The statistical analysis was performed using the SPSS software package (SPSS version 23.0.0, IBM Corporation, Armonk, NY, USA). Descriptive analyses were used for sociodemographic characteristics and to report the results obtained from the DT. *Student’s t* test or *Mann-Whitney U* test and analysis of variance (ANOVA) were used to analyze differences in mean values. Effect sizes were analyzed with *Cohen’s d*. *Spearman* correlation was performed to analyze the relationship between distress and age or parameters of tumor classification. All methods were carried out in accordance with relevant guidelines and regulations.

## Results

The total sample size was 120 patients, of which 83.3% were male. The mean age was 62.6 (SD 8.6) and ranged from 41 to 85 years. The patients presented with newly diagnosed (70, 58.3%), recurrent (13, 10.9%) or after (37, 30.8%) curative therapy of HNSCC. In all, fifty patients (41.7%) answered the questionnaire after they had received therapy for HNSCC in the past. Seventy-four patients (61.7%) were married or cohabited with a partner. The tumors were mainly located in the oropharynx (43.3%) followed by the larynx (21.7%) and oral cavity (20.8%). Twenty-seven patients (22.5%) received surgery only, 49 (40.8%) received surgery and adjuvant radio(chemo)therapy, 39 (32.5%) received curative radio(chemo)therapy and 5 patients (4.2%) underwent palliative radio(chemo)therapy. Chi-Squared-Test revealed a significant relationship among patients with distant metastases, as patients with distant metastases where significantly more likely to have a DTS ≥ 5. Further patient characteristics are shown in Table 1.

**Table 1:** Descriptive statistics and Chi-Squared-Test for patient characteristics

Age	Total	DTS < 5	DTS ≥ 5	DTS missing	***p***
Range	41–85	48–83	43–85	41–81	–
Mean	62.6	64.2	60.6	63.9	
Standard deviation	8.6	7.1	7.9	10.7	
**Gender**					
Male	100 (83.3%)	34 (28.4%)	42 (35.0%)	24 (20.0%)	.26
Female	20 (16.7%)	4 (3.3%)	10 (8.3%)	6 (5.0%)	
**Site**					
Oropharynx	52 (43.3%)	18 (15.0%)	24 (20.0%)	10 (8.3%)	.19
Larynx	26 (21.7%)	8 (6.7%)	10 (8.3%)	8 (6.7%)	
Oral cavity	25 (20.8%)	6 (5.0%)	9 (7.5%)	10 (8.3%)	
Hypopharynx	13 (10.8%)	6 (5.0%)	6 (5.0%)	1 (0.8%)	
Other	4 (3.4%)	0 (0.0%)	3 (2.5%)	1 (0.8%)	
**Stage/Metastases**					
I	17 (14.2%)	4 (3.3%)	9 (7.5%)	17 (14.2%)	.48
II	27 (22.5%)	13 (10.8%)	8 (6.7%)	13 (10.8%)	
III	24 (20.0%)	8 (6.7%)	8 (6.7%)	0	
IVa	39 (32.5%)	12 (10.0%)	18 (15.0%)	0	
IVb	10 (8.3%)	1 (0.8%)	6 (5.0%)	0	
IVc	3 (2.5%)	0	3 (2.5%)	0	
N0	49 (40.8%)	15 (12.5%)	22 (18.3%)	12 (10.0%)	.59
N1	18 (15.0%)	4 (3.3%)	10 (8.3%)	4 (3.3%)	
N2	41 (34.2%)	17 (14.2%)	16 (13.3%)	9 (7.5%)	
N3	12 (10.0%)	2 (1.7%)	4 (3.3%)	5 (4.1%)	
M0	113 (94.2%)	38 (31.7%)	47 (39.2%)	28 (23.3%)	**.048**
M1	7 (5.8%)	0 (0.0%)	5 (4.1%)	2 (1.7%)	
**Therapy**					
Surgery	27 (22.5%)	7 (18.5%)	12 (23.1%)	8 (6.7%)	.21
Surgery + adjuvant R(C)T	49 (40.8%)	17 (14.1%)	19 (15.8%)	13 (10.9%)	
Primary R(C)T	39 (32.5%)	14 (11.7%)	17 (14.1%)	8 (6.7%)	
Palliative R(C)T	5 (4.2%)	0 (0.0%)	4 (3.3%)	1 (0.8%)	

The mean DTS was 4.7 (*n* = 90, SD 2.4, 30 DTS missing). Fifty-two patients (57.7%) were at or above the cut-off score ≥ 5. Patients with newly diagnosed HNSCC (before therapy) had significantly higher distress scores (mean DTS 5.2, SD 2.2) than patients with recurrent HNSCC or after cure (mean DTS 4.1, SD 2.5, *p* = .032, *d* = .46). There were no differences between mean DTS for tumor sites (Welch-ANOVA: F(3, 32.3) = .75, *p* = .53), gender (*p* = .10), or relationship status (*p* = .99). No significant correlation was found between the DTS and age (*p* = .07), tumor size (*p* = .53) or presence of metastases in lymph nodes (*p* = .54).

Mann-Whitney *U*-test revealed no significant difference (*U* = 187.00, *p* = .31) in DTS between the two groups of patients with newly diagnosed HNSCC (47 DTS available) and recurrent disease only (10 DTS available).

Among patients with a DTS ≥ 5, 92.3% (48 patients) selected at least one item in the physical and 61.5% (28 patients) in the emotional domain. The rates of endorsement of at least one item for the other domains were 7.7% (4 patients) for the practical domain, 1.9% for the family domain (1 patient) and 3.8% (2 patients) for the spiritual domain (Table 2).

**Table 2:** Rates of endorsement for problem list items for all patients, patients with DTS < 5 and DTS ≥ 5

Problem list items	Total (n = 120)	DTS < 5 (***n*** = 38)	DTS ≥ 5 (***n*** = 52)	Missing (total)
**Practical domain**				
Housing	2.5% (3)	2.6% (1)	1.9% (1)	2.5% (3)
Insurance	5.8% (7)	7.9% (3)	1.9% (1)	0.8% (1)
Work/school	1.7% (2)	2.6% (1)	0.0%	3.3% (4)
Transportation	5.0% (6)	7.9% (3)	0.0%	1.7% (2)
Child care	0.8% (1)	2.6% (1)	0.0%	1.7% (2)
Financial problems	5.8% (7)	0.0%	5.8% (3)	0.8% (1)
**Family domain**				
Dealing with family members	2.5% (3)	2.6% (1)	0.0%	0.8% (1)
Dealing with children	3.3% (4)	2.6% (1)	1.9% (1)	1.7% (2)
Dealing with partner	2.5% (3)	0.0%	0.0%	2.5% (3)
**Emotional domain**				
Worries	29.2% (35)	21.1% (8)	38.5% (20)	2.5% (3)
Anxiety	35.0% (42)	23.7% (9)	46.2% (24)	0.8% (1)
Sadness	20.0% (24)	7.9% (3)	25.0% (13)	1.7% (2)
Depression	9.2% (11)	7.9% (3)	7.7% (4)	2.5% (3)
Nervousness	28.3% (34)	13.2% (5)	34.6% (18)	1.7% (2)
Loss of interests in usual activities	15.0% (18)	10.5% (4)	17.3% (9)	3.3% (4)
**Spiritual/ religious domain**				
Relating to God	1.7% (2)	0.0%	3.8% (2)	0.0%
Loss of faith	1.7% (2)	0.0%	3.8% (2)	0.0%
**Physical domain**				
Pain	34.2% (41)	34.2% (13)	44.2% (23)	1.7% (2)
Nausea	5.0% (6)	5.3% (2)	5.8% (3)	0.0%
Exhaustion	32.5% (39)	23.7% (9)	38.5% (20)	0.0%
Sleep	35.8% (43)	28.9% (11)	42.3% (22)	1.7% (2)
Mobility	30.8% (37)	23.7% (9)	42.3% (22)	0.8% (1)
Bathing/ dressing	6.7% (8)	5.1% (3)	3.8% (2)	0.0%
Appearance	6.7% (8)	2.6% (1)	5.8% (3)	0.8% (1)
Breathing	25.0% (30)	21.1% (8)	25.0% (13)	1.7% (2)
Mouth sores	46.7% (56)	26.3% (10)	57.7% (30)	0.8% (1)
Eating	34.2% (41)	28.9% (11)	36.5% (19)	0.0%
Indigestion/Constipation/ Diarrhea	9.2% (11)	2.6% (1)	9.6% (5)	0.8% (1)
Changes in Urination	0.0%	0.0%	0.0%	0.0%
Fever	13.3% (16)	7.9% (3)	17.3% (9)	0.8% (1)
Skin dry/ itchy	5.0% (6)	2.6% (1)	9.6% (5)	0.0%
Nose dry/ congested	5.0% (6)	7.9% (3)	5.8% (3)	0.0%
Tingling in hands/ feet	14.2% (17)	13.2% (5)	17.3% (9)	0.0%
Feeling swollen	8.3% (10)	7.9% (3)	7.7% (4)	0.0%
Memory/ concentration	11.7% (14)	5.3% (2)	11.5% (6)	0.0%
Sexual problems	6.7% (8)	5.3% (2)	7.7% (4)	3.3% (4)
**Demand for psycho-oncological support**	5.0% (6)	0.0%	9.6% (5)	13.3% (16)

There was a significant difference in the number of problem items selected between those with DTS ≥ 5 (mean number of items 2.6, SD 1.8) and those with DTS < 5 (mean number of items 1.6, SD 1.8, *p* = .014, *d* = .56). Furthermore, there was a significant positive correlation between DTS and the number of problem items selected (*p* < .001, *ρ* = .38).

**Fig. 2:**
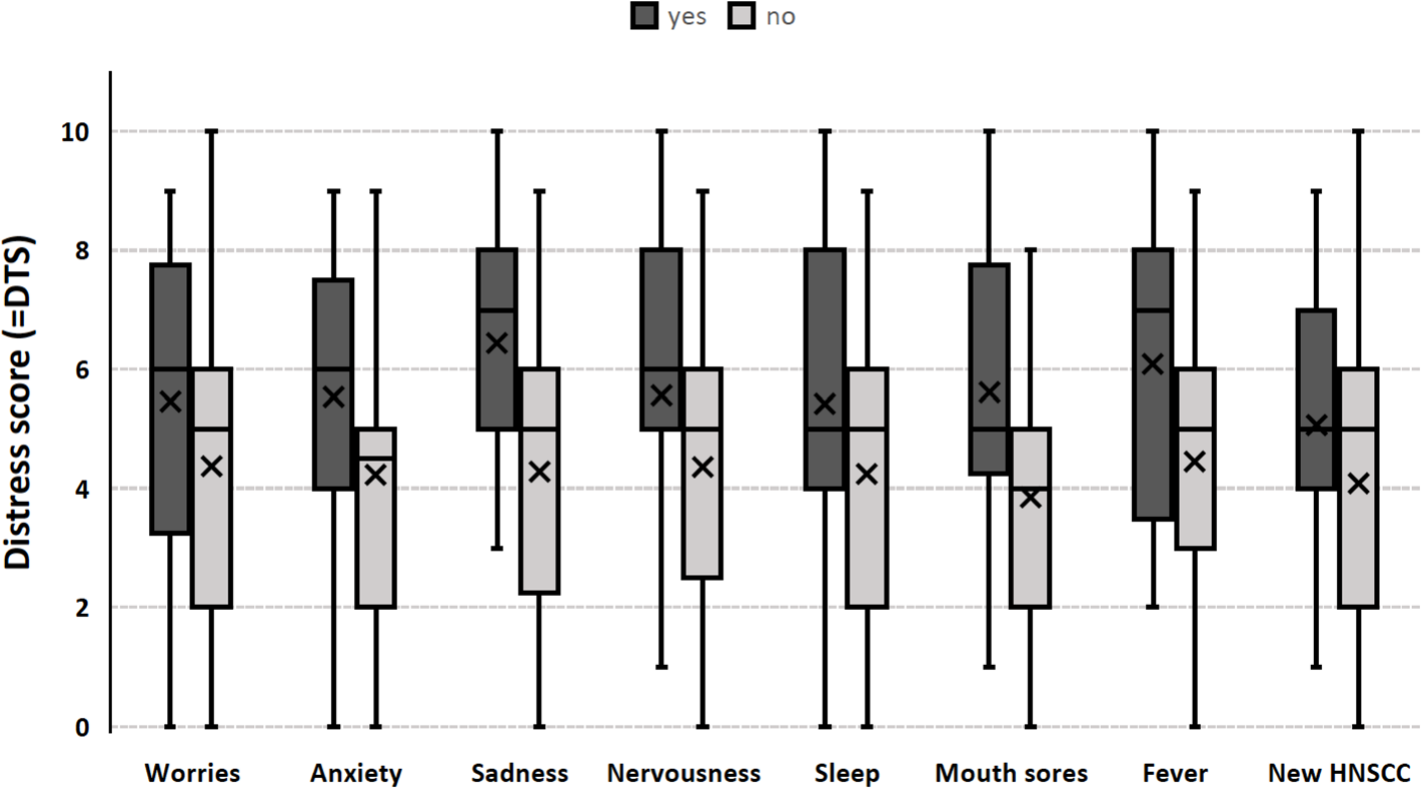
Grouped Box Plot (X = mean values) for DTS between selected problem items and newly diagnosed HNSCC

Patients who suffered from general worries, anxiety, sadness, nervousness, sleeping disorders, mouth sores, fever or newly diagnosed HNSCC had significantly higher DTS compared to individuals without these complaints (Table 3, Fig. 2). The calculation of effect sizes between these endorsed problem items showed a variety of medium effects (Table 3). To further investigate the influence of the significant relationship among patients with distant metastases (Table 1), we excluded these 7 patients for another analysis of differences in DTS mean values for patients, who indicated they suffered from the problems stated above.

**Table 3:** Significant differences (Mann-Whitney-U-Test) and effect sizes between DTS for selected problems items and newly diagnosed HNSCC

	Yes	No	n_**DTS**_	***p***	***d***
**Worries**	5.5 (SD 2.3, *n* = 28)	4.3 (SD 2.4, *n* = 61)	89 (1 missing)	.033	.46
**Anxiety**	5.5 (SD 2.3, *n* = 33)	4.2 (SD 2.3, *n* = 56)	89 (1 missing)	.008	.58
**Sadness**	6.4 (SD 2.6, *n* = 16)	4.3 (SD 2.2, *n* = 72)	88 (2 missing)	<.001	.42
**Nervousness**	5.6 (SD 2.5, *n* = 23)	4.4 (SD 2.3, *n* = 65)	88 (2 missing)	.028	.48
**Sleep**	5.4 (SD 2.4, n = 33)	4.3 (SD 2.3, n = 56)	89 (1 missing)	.042	.42
**Mouth sores**	5.6 (SD 2.1, *n* = 40)	3.9 (SD 2.3, *n* = 49)	89 (1 missing)	<.001	.76
**Fever**	6.1 (SD 2.7, n = 12)	4.5 (SD 2.3, *n* = 77)	89 (1 missing)	.042	.44
**New HNSCC**	5.2 (SD 2.2, *n* = 47)	4.1 (SD 2.5, *n* = 43)	90 (0 missing)	.032	.46

**Fig. 3:**
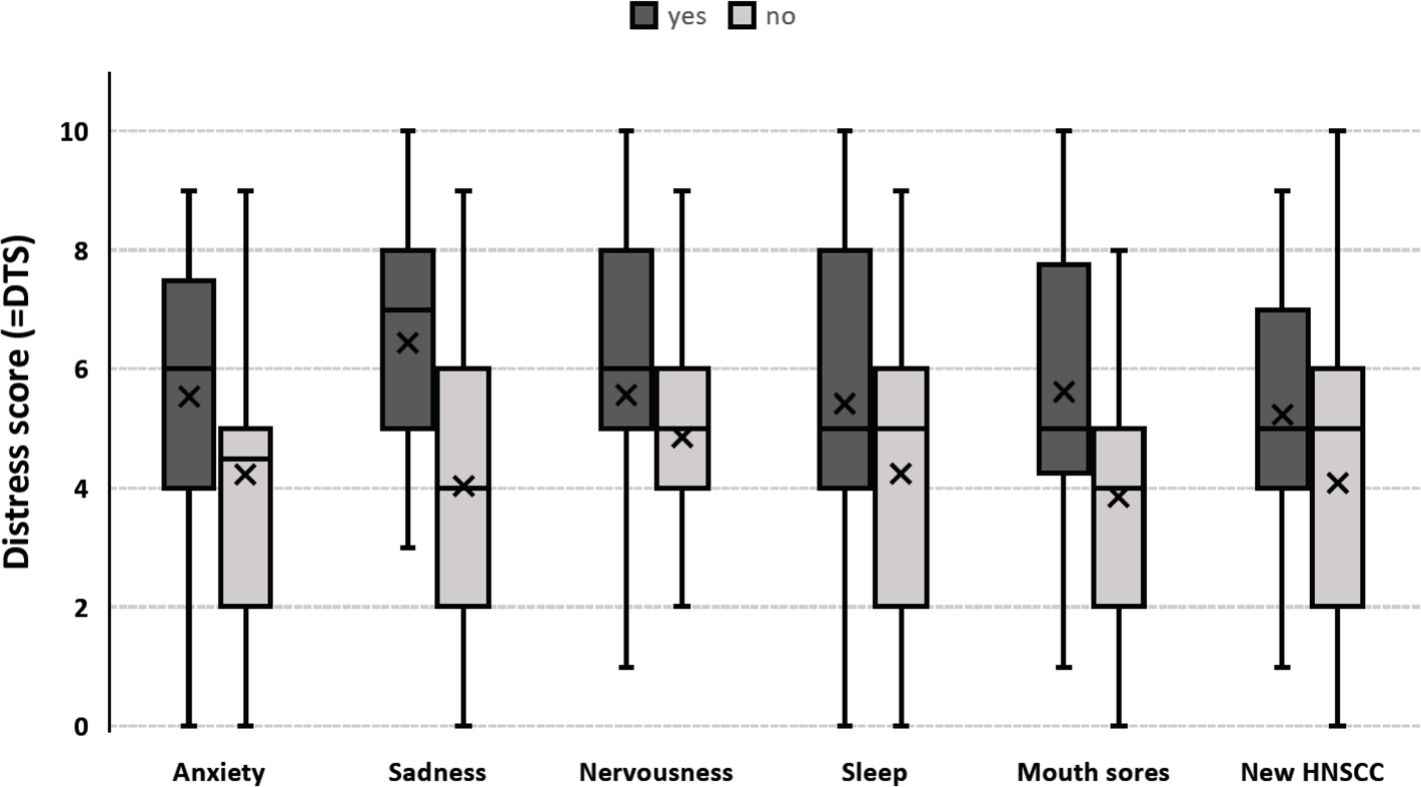
Grouped Box Plot for DTS (X = mean values) between selected problem items and newly diagnosed HNSCC (patients with distant metastases excluded)

After excluding these individuals, the mean DTS was 4.6 (SD 2.5). The calculation showed significantly higher DTS, compared to individuals without these complaints, for anxiety, sadness, nervousness, sleeping disorders, mouth sores and newly diagnosed HNSCC (Table 4, Fig. 3). The calculation of effect sizes between these endorsed problem items also showed a variety of medium effects. After exclusion of patients with distant metastases the differences between DTS mean values in patients, who suffered from general worries or fever, were no longer significant.

**Table 4:** Significant differences (Mann-Whitney-U-Test) and effect sizes between DTS vs. selected problem items and newly diagnosed HNSCC (patients with distant metastases excluded)

	Yes	No	n_**DTS**_	***p***	***d***
**Anxiety**	5.4 (SD 2.4, *n* = 31)	4.1 (SD 2.4, *n* = 53)	84 (2 missing)	.011	.56
**Sadness**	6.5 (SD 2.7, *n* = 15)	4.2 (SD 2.2, *n* = 68)	83 (2 missing)	.002	.72
**Nervousness**	5.5 (SD 2.5, *n* = 22)	4.3 (SD 2.4, n = 61)	83 (2 missing)	.028	.49
**Sleep**	5.3 (SD 2.5, n = 31)	4.2 (SD 2.4, n = 53)	84 (1 missing)	.045	.44
**Mouth sores**	5.6 (SD 2.3, *n* = 36)	3.9 (SD 2.3, *n* = 48)	84 (1 missing)	.002	.72
**New HNSCC**	5.2 (SD 2.2, n = 47)	3.8 (SD 2.5, n = 38)	85 (0 missing)	.010	.57

Psychological support was demanded by 6 individuals out of a total of 104 patients (5.0%, 16 missing). Out of those 6 individuals, 5 had a DTS ≥ 5 (1 DTS missing).

## Discussion

Coping with diagnosis and outcome of treatment means a significant physical and psychological burden for HNSCC patients, especially as HNSCC and its treatment may result in disfigurement and dysfunction. Unmet psychosocial needs can complicate the treatment course and recovery of HNSCC patients as well as their survival [24]. In the absence of distress screening, distress may remain unrecognized or underestimated by clinicians in the often very busy oncology settings. In a sample of German patients with oral squamous cell carcinoma, Schell et al. [4] detected a mean DTS of 5.7 with 72% of the patients with a DTS ≥ 5. In a study from Taiwan, oral cancer patients, on average, had only moderate distress levels (mean DTS 2.27). Based on previous studies, a DTS of ≥ 5 represents the threshold for those patients who will benefit from psychological support [25]. The present study revealed that among HNSCC patients, screening for distress seems to be valuable since 57.7% of our cohort were screened positive for significant distress and the mean DTS was 4.7, SD 2.4. A previous study from the United Kingdom gave a lower significant distress rate (31%) in head and neck cancer patients [10]. Interestingly, there might be an impact of knowing about a better outcome [26] in p16+ (HPV-driven) OPSCC patients as this group, according to the lower mean DTS of 1.5 reported by the 10 patients available versus mean DTS of 5.8 in the p16- OPSCC patients, reported lower levels of distress. HPV status could be one important factor explaining less distress in p16+ OPSCC patients, not only for the perceived better prognosis, but also for the different social and cultural environment [5], from which these patients might benefit. Based on the small number of p16+ OPSCC patients, we were unable to further investigate this possibly important link in our cohort.

Patients in our study showed high levels of unmet needs in the emotional and physical domain. In the emotional domain, the present analysis confirms the importance of anxiety and worries as common problems in about two thirds of the patients. In the physical domain “mouth sores” was the most often mentioned problem (46.7%), followed by “sleep” (35.8%) and “pain” (34.2%), which also were the most often selected items in the patient group with significant distress. Similar to the study of Schell et al. the DTS neither correlated with age, nor with tumor size [4]. Furthermore, Ghazali et al. were recently able to show that significant distress was associated with poor health-related quality of life in a group of HNC survivors [10].

In our cohort, patients with significant distress chose a significantly higher number of problems on the items list than patients with a DTS < 5. The total number of selected items also showed a significant positive correlation with the DTS. An effect size of *d* = .56 furthermore indicates a relevant effect for these findings [27], whereas the correlation coefficient *ρ* = .38 shows a moderate correlation between these variables. In combination however, these results suggest that individuals, who tend to report more problems, also have higher distress or vice versa. Future prospective studies could investigate whether the number of selected items on the problems list predicts patients’ distress as valid as the DTS itself. On this basis, a second cut-off score could possibly be established to include the problems list for the evaluation of the distress thermometer, making the evaluation process more independent of the DTS alone.

Patients who chose the items “mouth sores”, “worries”, “anxiety”, “sadness, “nervousness”, “fever” and “sleep” on the problems list showed significantly higher DTS than patients, who did not report these problems. Patients selecting “sadness” as a problem, interestingly showed the highest mean DTS (6.3, SD 2.8). The highest effect size *d* = .76 was found for patients, who stated “mouth sores” as problem, which has to be interpreted as a relevant effect [27]. These findings possibly indicate the importance of these particular items for the patient’s current psycho-emotional health status and suggest that physicians may need to pay special attention, whenever patients endorse them. Relevant effects [27] were also found for “anxiety”, “nervousness” and “fever”. These findings might emphasize the importance of acknowledging and handling these specific problems, whenever the patients report them. Patients with newly diagnosed HNSCC before therapy showed significantly higher DTS (5.2, SD 2.2) and a relevant effect size *d* = .46 [27], compared to patients, who received therapy in the past (DTS 4.1, SD 2.5). Our results confirm the findings of previous studies that found significant DTS of HNSCC patients before clinical assessment and therapy [12]. However, our cohort showed relatively high DTS, compared to other studies that found, for example, a mean DTS of 2.9 for head and neck cancer patients two years (median) after therapy [10]. The best explanation might be the lower DTS after curative treatment we observed in our cohort, too. Exclusion of patients with distant metastases did not relevantly influence the mean DTS (4.6, SD 2.5) of our sample. After exclusion of these patients, significant differences in mean DTS were also found for “anxiety”, “sadness”, “nervousness”, “sleep”, “mouth sores” and patients with newly diagnosed HNSCC, but no longer for “worries” or “fever”. Overall, the significant relationship between patients with distant metastases and elevated distress levels in the total sample neither seems to influence the mean DTS in our total cohort, nor for individuals with a DTS ≥ 5 and the selected problems above in a relevant way, as similar effect sizes were found after exclusion of these patients. Many HNSSC patients, who report high levels of distress, are not trying to take advantage of psycho-oncological care [28]. Various studies demonstrated that distress did not, or only moderately correlate with the wish for psycho-oncological support provided by specialists [7, 29, 30]. Cancer patients who accept psycho-oncological care typically are rather young [31–33], female [29, 34] and present higher levels of education [34, 35]. In our patient group, despite a mean DTS of 4.7 and fifty-two patients with significant distress, only 6 out of 104 patients (5.8%) desired psycho-oncological support. These results clearly support the findings of previous studies, which did not find a correlation between distress and the explicit wish for psycho-oncological support from professionals [29] potentially indicating a lack of awareness for the importance of psycho-oncological support in these patients. This can be caused by various reasons, for example unawareness about potential benefits and no subjective need for psycho-oncological treatment in these patients. Further barriers could be an inadequate knowledge of psycho-oncological services and an ineffective patient-physician-communication, which does not encourage patients to desire psycho-oncological treatment. In some cases, there is also a reluctance by patients to be referred to psychosocial support due to expected stigmatization. Even patients who score high on the DT may not necessarily want help. Other studies showed high referral rates when psycho-oncologists visit distressed patients mandatorily [36]. The present results emphasize the need to inform patients about the availability of psycho-oncological support and its potential benefits, as for instance, previous studies were able to show the detrimental effects of distress on health in patients with chronic disease [37]. It also has to be ensured that physicians systematically act on DT screening results as a DTS ≥ 5 should be managed professionally by following clinical assessment. Previous studies demonstrated that when patients were screened and did not receive any referrals or psycho-oncological assistance, their levels of distress increased [36]. However, we found lower distress levels in patients after treatment although most of them did not have psycho-oncological support. These patients might have had enough time or other support than psycho-oncological therapy to deal with their cancer diagnosis during the process of therapy. On the other hand, in breast cancer patients, it could be demonstrated that psychologic intervention does improve survival [38]. In HNSCC patients and other tumor entities evidence is still pending that psycho-oncological treatment with lowering of distress can lead to better survival. A meta-analysis on the impact of psychosocial intervention on survival in cancer revealed that group-delivered psychosocial interventions demonstrated only short-term improvements in survival and individually-delivered interventions even failed to show any survival benefit [39].

### Limitations

Given the retrospective character of the acquired data there are several limitations of this study. It consists of a relatively small number of patients and there was variability in time elapsed since cancer-diagnosis. Inherent to the design we have to deal with some missing data, which could have impacted the presented results. We cannot provide information about the presence or absence of a caregiver, which could possibly have influenced patients DTS, as this information is not routinely assessed. Any ongoing care for the HNSCC patients by a psychologist independent from our hospital cannot be excluded as we did not ask explicitly for this potentially relevant factor that consequently could not be considered in the data analysis.

## Conclusion

HNSCC patients suffer from high distress levels, but the HNSCC patients’ supportive care needs must be routinely assessed to provide a basis to better understand how distress management can be improved. In the present study, HNSCC patients, who suffered from newly diagnosed HNSCC and sadness, general worries, anxiety, nervousness, sleeping disorders, mouth sores or fever on the problem list of the DT were at higher risk for significant distress compared to HNSCC patients without those complaints. Distress screening enhances the awareness of psycho-oncological issues, but its use in HNSCC patients is associated with some difficulties as only a small percentage of our cohort, regardless of being at the stage before or after therapy, desired psycho-oncological support. Further research is needed to investigate to what extent and at which point of time HNSCC patients desire psychosocial care and which activities raise the acceptance of psycho-oncological support in these patients. Addressing HNSCC patients’ supportive care needs by offering visiting a psycho-oncologist routinely to each patient might be a first step to do so.

## Data Availability

The datasets used and/or analyzed during the current study are available from the corresponding author on reasonable request.

## Abbreviations

*ANOVA*: Analysis of variance
*d*: Cohen’s d (measure for effect size)
*DT*: Distress Thermometer
*DTS*: Distress Thermometer score (on the visual analogue scale)
*HNC*: Head and neck cancer
*HNSCC*: Head and neck squamous cell carcinoma
*HPV*: Human papilloma virus
*n*: Sample size
*NCCN*: National Comprehensive Cancer Network
*OPSCC*: Oropharyngeal squamous cell carcinoma
*p*: *p*-Value
*p16*: Protein for diagnosis of HPV infected cells via immunohistochemistry
*ρ*: Spearman’s Rho (correlation coefficient)
*SD*: Standard deviation
*SPSS*: Statistical package for the Social Sciences
*TNM*: Tumor (T), nodes (N) and metastases (M). Classification system of malignant tumors

## References

[1] Fischer DJ, Villines D, Kim YO, Epstein JB, Wilkie DJ (2010). Anxiety, depression, and pain: differences by primary cancer. Support Care Cancer.

[2] Mehnert A, Brähler E, Faller H, Härter M, Keller M, Schulz H, Wegscheider K, Weis J, Boehncke A, Hund B, Reuter K, Richard M, Sehner S, Sommerfeldt S, Szalai C, Wittchen HU, Koch U (2014). Four-week prevalence of mental disorders in patients with cancer across major tumor entities. J Clin Oncol.

[3] Anguiano L, Mayer DK, Piven ML, Rosenstein D (2012). A literature review of suicide in cancer patients. Cancer Nurs.

[4] Schell J-T, Petermann-Meyer A, Kloss-Brandstätter A, Bartella AK, Kamal M, Hölzle F, Lethaus B, Teichmann J (2018). Distress thermometer for preoperative screening of patients with oral squamous cell carcinoma. J Craniomaxillofac Surg.

[5] Dodd RH, Forster AS, Marlow LAV, Waller J (2019). Psychosocial impact of human papillomavirus-related head and neck cancer on patients and their partners: A qualitative interview study. Eur J Cancer Care (Engl).

[6] Lee J-Y, Jung D, Kim W-H, Lee H-J, Noh D-Y, Hahm B-J (2016). Correlates of oncologist-issued referrals for psycho-oncology services: what we learned from the electronic voluntary screening and referral system for depression (eVSRS-D). Psycho-oncology..

[7] Ernst J, Faller H, Koch U, Brähler E, Härter M, Schulz H, Weis J, Köhler N, Hinz A, Mehnert A (2018). Doctor's recommendations for psychosocial care: frequency and predictors of recommendations and referrals. PLoS One.

[8] Schweer C, Doering S, Haier J, Heuft G, Fritz F, Dugas M, Schneider G (2011). Psychoonkologische Angebote - was wunschen uber 60-jahrige Krebspatienten?. Z Psychosom Med Psychother.

[9] Roth AJ, Kornblith AB, Batel-Copel L, Peabody E, Scher HI, Holland JC (1998). Rapid screening for psychologic distress in men with prostate carcinoma. Cancer..

[10] Ghazali N, Roe B, Lowe D, Tandon S, Jones T, Brown J, Shaw R, Risk J, Rogers SN (2017). Screening for distress using the distress thermometer and the University of Washington Quality of life in post-treatment head and neck cancer survivors. Eur Arch Otorhinolaryngol.

[11] Lehmann-Laue A, Danker H, Schröter K, Friedrich M, Mehnert A, Ernst J (2019). Psychosoziale Versorgung von Krebspatienten in einer Krebsberatungsstelle an einem Universitätsklinikum. Psychother Psychosom Med Psychol.

[12] Maher NG, Britton B, Hoffman GR (2013). Early screening in patients with head and neck cancer identified high levels of pain and distress. J Oral Maxillofac Surg.

[13] Buchmann L, Conlee J, Hunt J, Agarwal J, White S (2013). Psychosocial distress is prevalent in head and neck cancer patients. Laryngoscope..

[14] Johnson C, George M, Fader AN (2017). Distress screening: evaluating a protocol for gynecologic Cancer survivors. Clin J Oncol Nurs.

[15] Dabrowski M, Boucher K, Ward JH, Lovell MM, Sandre A, Bloch J, Carlquist L, Porter M, Norman L, Buys SS (2007). Clinical experience with the NCCN distress thermometer in breast cancer patients. J Natl Compr Cancer Netw.

[16] de Mol M, den Oudsten BL, Aarts M, Aerts JGJV (2017). The distress thermometer as a predictor for survival in stage III lung cancer patients treated with chemotherapy. Oncotarget.

[17] Mehnert A, Müller D, Lehmann C, Koch U (2006). Die deutsche Version des NCCN Distress-Thermometers. Z Psychiatr Psychol Psychother.

[18] Mitchell AJ (2010). Short screening tools for cancer-related distress: a review and diagnostic validity meta-analysis. J Natl Compr Cancer Netw.

[19] O'Donnell E (2013). The distress thermometer: a rapid and effective tool for the oncology social worker. Int J Health Care Qual Assur.

[20] Carlson LE, Zelinski EL, Toivonen KI, Sundstrom L, Jobin CT, Damaskos P, Zebrack B (2019). Prevalence of psychosocial distress in cancer patients across 55 north American cancer centers. J Psychosoc Oncol.

[21] Heyes SM, Bond MJ, Harrington A, Belan I (2016). The relative contributions of function, perceived psychological burden and partner support to cognitive distress in bladder cancer. Psycho-oncology..

[22] Arden-Close E, Gidron Y, Moss-Morris R (2008). Psychological distress and its correlates in ovarian cancer: a systematic review. Psycho-oncology..

[23] Kim GM, Kim SJ, Song SK, Kim HR, Kang BD, Noh SH, Chung HC, Kim KR, Rha SY (2017). Prevalence and prognostic implications of psychological distress in patients with gastric cancer. BMC Cancer.

[24] Chen AM, Hsu S, Felix C, Garst J, Yoshizaki T (2018). Effect of psychosocial distress on outcome for head and neck cancer patients undergoing radiation. Laryngoscope..

[25] Jacobsen PB, Donovan KA, Trask PC, Fleishman SB, Zabora J, Baker F, Holland JC (2005). Screening for psychologic distress in ambulatory cancer patients. Cancer..

[26] Du E, Mazul AL, Farquhar D, Brennan P, Anantharaman D, Abedi-Ardekani B (2019). Long-term survival in head and neck Cancer: impact of site, stage, smoking, and human papillomavirus status. Laryngoscope..

[27] Hattie J (2010). Visible learning: a synthesis of over 800 meta-analyses relating to achievement.

[28] Verdonck-de Leeuw IM, de Bree R, Keizer AL, Houffelaar T, Cuijpers P, van der Linden MH, Leemans CR (2009). Computerized prospective screening for high levels of emotional distress in head and neck cancer patients and referral rate to psychosocial care. Oral Oncol.

[29] Merckaert I, Libert Y, Messin S, Milani M, Slachmuylder JL, Razavi D (2010). Cancer patients' desire for psychological support: prevalence and implications for screening patients' psychological needs. Psycho-oncology.

[30] Faller H, Weis J, Koch U, Brähler E, Härter M, Keller M, Schulz H, Wegscheider K, Boehncke A, Hund B, Reuter K, Richard M, Sehner S, Wittchen HU, Mehnert A (2017). Utilization of professional psychological care in a large German sample of cancer patients. Psycho-oncology..

[31] Tuinman MA, Gazendam-Donofrio SM, Hoekstra-Weebers JE (2008). Screening and referral for psychosocial distress in oncologic practice: use of the distress thermometer. Cancer..

[32] Ellis J, Lin J, Walsh A, Lo C, Shepherd FA, Moore M, Li M, Gagliese L, Zimmermann C, Rodin G (2009). Predictors of referral for specialized psychosocial oncology care in patients with metastatic cancer: the contributions of age, distress, and marital status. J Clin Oncol.

[33] Zeissig SR, Singer S, Koch L, Blettner M, Arndt V (2015). Inanspruchnahme psychoonkologischer Versorgung im Krankenhaus und in Krebsberatungsstellen durch Brust-, Darm- und Prostatakrebsüberlebende. Psychother Psychosom Med Psychol.

[34] Nekolaichuk CL, Cumming C, Turner J, Yushchyshyn A, Sela R (2011). Referral patterns and psychosocial distress in cancer patients accessing a psycho-oncology counseling service. Psycho-oncology..

[35] Mehnert A, Koch U (2005). Psychosocial care of cancer patients--international differences in definition, healthcare structures, and therapeutic approaches. Support Care Cancer.

[36] Mitchell AJ (2013). Screening for cancer-related distress: when is implementation successful and when is it unsuccessful?. Acta Oncol.

[37] Barry V, Stout ME, Lynch ME, Mattis S, Tran DQ, Antun A, Ribeiro MJA, Stein SF, Kempton CL (2020). The effect of psychological distress on health outcomes: a systematic review and meta-analysis of prospective studies. J Health Psychol.

[38] Andersen BL, Yang H-C, Farrar WB, Golden-Kreutz DM, Emery CF, Thornton LM, Young DC, Carson WE (2008). Psychologic intervention improves survival for breast cancer patients: a randomized clinical trial. Cancer..

[39] Fu WW, Popovic M, Agarwal A, Milakovic M, Fu TS, McDonald R (2016). The impact of psychosocial intervention on survival in cancer: a meta-analysis. Ann Palliative Med.

